# Changing Patterns of Gastrointestinal Parasite Infections in Cambodian Children: 2006–2011

**DOI:** 10.1093/tropej/fms024

**Published:** 2012-06-18

**Authors:** Catrin E. Moore, Put Chhat Hor, Sona Soeng, Sopheary Sun, Sue J. Lee, Christopher M. Parry, Nicholas P. J. Day, Nicole Stoesser

**Affiliations:** ^1^Mahidol-Oxford Tropical Medicine Research Unit, Faculty of Tropical Medicine, Mahidol University, 420/6 Ratchawithi Road, Ratchathewi, Bangkok 10400, Thailand; ^2^Departments of Microbiology and Information Technology, Angkor Hospital for Children, Siem Reap, Kingdom of Cambodia; ^3^Nuffield Department of Clinical Medicine, Center for Clinical Vaccinology and Tropical Medicine, University of Oxford, Churchill Hospital, Oxford OX3 7LJ, UK

**Keywords:** Cambodia, parasite, faeces, paediatric, epidemiology

## Abstract

We studied gastrointestinal parasites in symptomatic Cambodian children attending a provincial hospital in Siem Reap, Cambodia between 2006 and 2011. A total of 16 372 faecal samples were examined by direct microscopy. Parasites were detected in 3121 (19.1%) samples and most common were *Giardia lamblia* (8.0% of samples; 47.6% disease episodes), hookworm (5.1%; 30.3%) and *Strongyloides stercoralis* (2.6%; 15.6%). The proportion of infected children increased, and the number of disease episodes effectively treated with a single dose of mebendazole decreased, over the 5-year period.

Intestinal helminths are a major global public health concern, particularly in developing countries [1]. Approximately 1.2 billion people in the Asia-Pacific region are infected with soil-transmitted helminths (STH) and/or schistosomes. Data for food-borne trematodes and cestodes are scarcer [2]. Paediatric helminth infections have detrimental effects on nutrition, growth and cognition and contribute to childhood anaemia [1].

Cambodia has some of the poorest health indicators in Southeast Asia, with high rates of malnutrition [3]. Less than one-third of the population have access to improved sanitation facilities and clean drinking water [3, 4]. Surveys in Cambodian children in 2000–2004 demonstrating a prevalence of STH infections of 25–52% [1, 5] led to the implementation of a national deworming initiative. Since 2006, single-dose mebendazole (500 mg) has been given twice yearly to school-aged (6–12 years) and pre-school children (6–59 months) [4]. Data on the impact of this programme remain limited [6, 7].

Angkor Hospital for Children (AHC) is a government provincial children’s hospital providing free medical care to children aged 0–16 years from Siem Reap and surrounding provinces. We studied faecal samples routinely submitted from children attending AHC between January 2006 and September 2011. Indications for sampling were diarrhoea, unexplained abdominal pain, malnutrition and/or anaemia. Direct microscopy of a wet preparation of faeces was undertaken with a modified Ziehl–Neelsen stain for *Cryptosporidium* spp. if requested. Data were extracted retrospectively from the hospital’s database. Sample results from a patient within a 2-month window-period were defined as a single disease episode. Proportions were compared using chi-squared, Fisher’s exact and Mantel–Haenszel (MH) tests (adjusted for month each year) as appropriate using Stata v10.0 (College Station, Texas, USA). Ethical approval was given by the AHC Institutional Review Board.

Of 16 372 faecal samples analysed, 12 622 (77.1%) were single samples from individuals and 3750 (22.9%) were multiple samples. In the 2756 disease episodes associated with gastrointestinal parasites, 2447 (88.8%) featured a single parasite type, 272 (9.9%) two types and 37 (1.3%) three types. Table 1 shows the parasites detected overall and by age group. There was no difference between genders (*p* = 0.63) but the proportion of positive samples varied significantly by age (chi-squared *p* < 0.0001). *Giardia lamblia* (*n* = 1311, 47.6% disease episodes), hookworm (*n* = 835, 30.3%) and *Strongyloides stercoralis* (*n* = 429, 15.6%) were most common. In 309 children with mixed infections, hookworm*/S. stercoralis* (*n* = 111, 35.9%), hookworm*/G. lamblia* (*n* = 65, 21.0%) and *G. lamblia/S. stercoralis* (*n* = 56, 18.1%) infections were predominant and all three parasites were found in 16 (5.2%) children.

**Table 1:** Gastrointestinal parasites identified in faecal samples from children in Siem Reap and surrounding provinces, January 2006–September 2011

	Total number *n* = 16 372 (% total)	Under 1 year *n* = 4738 (%^a^)	1–5 years *n* = 6439 (%^a^)	6–10 years *n* = 2748 (%^a^)	11–16 years *n* = 2447 (%^a^)
Protozoa					
*Giardia lamblia*	1311 (8.0)	59 (67.0)	709 (53.4)	316 (34.0)	227 (29.4)
*Blastocystis hominis*	180 (1.1)	5 (5.7)	66 (5.0)	50 (5.4)	59 (7.6)
*Entamoeba histolytica*	97 (0.7)	5 (5.7)	36 (2.7)	33 (3.6)	23 (3.0)
*Cryptosporidium parvum*	3 (0.02)	0	2 (0.2)	1 (0.1)	0
Helminths					
Nematodes					
Hookworm	835 (5.1)	7 (8.0)	267 (20.1)	290 (31.2)	271 (34.8)
*Strongyloides stercoralis*	429 (2.6)	9 (10.2)	149 (11.2)	140 (15.1)	131 (16.8)
*Enterobius vermicularis*	51 (0.3)	1 (1.1)	23 (1.7)	17 (1.8)	10 (1.3)
*Ascaris lumbricoides*	41 (0.3)	0	20 (1.5)	12 (1.3)	9 (1.2)
*Trichuris trichiura*	9 (0.05)	0	5 (0.4)	3 (0.3)	2 (0.1)
Cestodes					
*Hymenolepis nana*	88 (0.5)	0	23 (1.7)	39 (4.2)	26 (3.3)
*Taenia saginata*	10 (0.06)	0	5 (0.4)	1 (0.1)	4 (0.5)
*Taenia solium*	24 (0.2)	1 (1.1)	13 (1.0)	9 (1.0)	1 (0.1)
*Taenia* species	6 (0.04)	0	0	4 (0.4)	2 (0.2)
*Hymenolepis diminuta*	2 (0.01)	0	0	0	2 (0.2)
Trematodes					
*Opisthorchis/Clonorchis* species	14 (0.09)	0	0	6 (0.6)	8 (1.0)
*Schistosoma* species	5 (0.03)	0	3 (0.2)	2 (0.2)	0
*Fasciola hepatica*	3 (0.02)	0	2 (0.2)	1 (0.1)	0
Unidentified parasite	13 (0.08)	1 (1.1)	4 (0.3)	4 (0.4)	4 (0.5)
Total	3121 (2756^b^)	88 (83^b^; 3.0%^c^)	1327 (1196^b^; 43.4%^c^)	928 (799^b^; 29.0%^c^)	779 (678^b^; 24.6%^c^)

^a^Percentage of those positive by age group (either < 1 year, 1–5 years, 6–10 years or 11–15 years).

^b^Number of disease episodes.

^c^Percentage of disease episode by age (number in age group/total number of episodes).

There was an overall increase in the proportion of infected children over the whole 5-year period (compared with negative samples) [MH adjusted OR 1.03 for all years (95% CI 1.01–1.04); Fig. 1]. Increases occurred for *G. lamblia* [MH OR 1.03, all years (95% CI 1.01–1.05)] and hookworm [MH OR 1.04, all years (95% CI 1.02–1.07)] but there was no significant change for *S. stercoralis* [MH OR 1.01, all years (95% CI 0.98–1.04); Fig. 1]. No seasonal variation was detected for common parasites (examined by month each year).

**Fig. 1.**
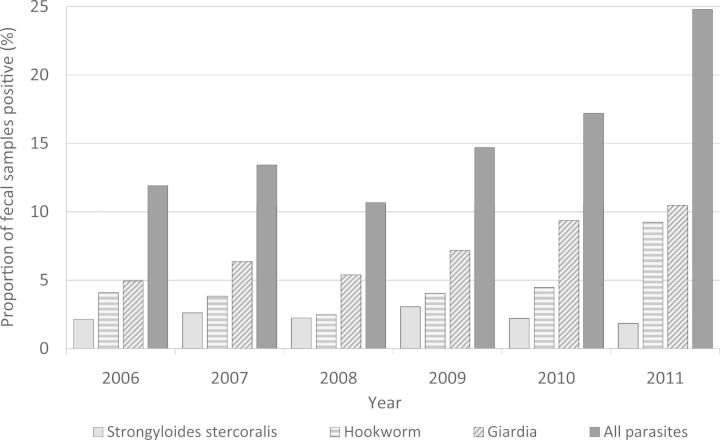
Percent proportion of the faecal parasites which were positive and the three most common parasites (*G. lamblia*, hookworm and *S. stercoralis*) in faecal samples over the 5-year study period (January 2006–September 2011).

Single-dose mebendazole has cure rates of ≥90% for only two of the parasites identified (*Ascaris* and *Enterobius*) [8, 9]. Using this percentage cure rate, only 91/2756 (3.3%) disease episodes would have been treated effectively by mebendazole, although it is possible an additional number of infections may have been cured. Of children whose infections would have been covered by mebendazole, 22 (24%) occurred in those too young for the deworming programmes. The number of children infected by parasites not covered by single-dose mebendazole increased significantly between 2008 and 2011 (*p* < 0.0001).

This first published report of gastrointestinal parasitic infections in symptomatic children from Siem Reap Province in Cambodia suggests a growing problem. There were annual increases of 1–4% for *G. lamblia* and hookworm infections although the number of *S. stercoralis* infections remained stable. Laboratory staff and methods remained unchanged during the study period as did the indications for sampling, with no significant differences in the proportion of patients attending the hospital sampled and no change in their age distribution. It is impossible to assess whether an increased awareness of parasitic diseases and a concomitant change in health-seeking behaviour occurred during the study.

Single-dose mebendazole has limited efficacy against *G. lamblia*, hookworm and *S. stercoralis* [8, 9] and the national deworming programme may also miss children not attending school. In our region, only 18–50% (depending on age group) of school-aged children regularly attend school (D. Vagni, unpublished results). Increases in giardiasis have been described following community-based deworming, and a ‘replacement’ effect is plausible [10]. Increasing numbers of hookworm infections highlight concerns about the potential emergence of resistance to anti-helminthics in humans and the lack of effective tools to monitor this.

An important limitation of this study is that direct microscopy was the only method used for parasite detection and there was no external quality control programme. This is the method currently in use in most hospitals in the resource-limited diagnostic setting of Cambodia and national estimates of parasite burden may be under-representative. Concentration techniques and/or agar plate cultures would have increased parasite detection [11]. A further limitation is the lack of detailed clinical information.

Systematic and prospective surveillance of faecal parasite infections in Cambodian children could help confirm if these observed increases are real and provide a baseline to monitor the impact of future interventions. Alternative approaches might include mass drug administration with other anti-parasitic drugs, such as albendazole (improved hookworm treatment) [8] and/or tinidazole (improved giardia treatment) and community-directed treatment where school attendance is limited [12]. An assessment of adult helminth infections in the region would be of interest [13]. Public health education and initiatives improving access to clean water and sanitation remain a priority.

## Funding

Funding for the study was provided by the Wellcome Trust of Great Britain and the Li Ka Shing-University of Oxford Global Health Program.
